# Ear transplantations reveal conservation of inner ear afferent pathfinding cues

**DOI:** 10.1038/s41598-018-31952-y

**Published:** 2018-09-14

**Authors:** Karen L. Elliott, Bernd Fritzsch

**Affiliations:** Department of Biology, University of Iowa, Iowa City, IA 52242 USA

## Abstract

Vertebrate inner ear neurons project into the correct brainstem nuclei region before target neurons become postmitotic, or even in their absence. Moreover, afferents from transplanted ears in frogs have been shown to navigate to vestibular nuclei, suggesting that ear afferents use molecular cues to find their target. We performed heterochronic, xenoplastic, and heterotopic transplantations in chickens to investigate whether inner ear afferents are guided by conserved guidance molecules. We show that inner ear afferents can navigate to the vestibular nuclei following a delay in afferent entry and when the ear was from a different species, the mouse. These data suggest that guidance molecules are expressed for some time and are conserved across amniotes. In addition, we show that chicken ears transplanted adjacent to the spinal cord project dorsally like in the hindbrain. These results suggest that inner ear afferents navigate to the correct dorsoventral brainstem column using conserved cues.

## Introduction

Inner ear sensory neurons are derived from the otic placode and make stereotyped connections with both inner ear hair cells at the periphery and second order central neuron targets in the hindbrain^1–3^. These neurons transmit vestibular and auditory information to the appropriate second order neurons in the hindbrain for normal function^4^. Within the hindbrain, inner ear auditory and vestibular sensory afferents project directly to the auditory and vestibular nuclei, respectively^5,6^. Vestibular afferents enter the hindbrain in Rhombomere 4 at the level of the lateral vestibular (aka Deiters) nucleus, bifurcate, and send an ascending branch to the superior vestibular nucleus and the cerebellum and a descending branch to the medial and inferior vestibular nuclei^7^. Auditory afferents enter the cochlear nucleus at Rhombomere 4, bifurcate, and send axons to the anteroventral cochlear nucleus, the posteroventral, and the dorsal cochlear nucleus in mice or to the nucleus magnocellularis and nucleus angularis in chicken^6,8,9^. While much is known about where inner ear afferents project to within the hindbrain, the mechanisms that guide auditory and vestibular afferents during development to project to their respective target nuclei within the hindbrain remain largely unknown.

During development, afferents from various neural crest and placode derived ganglia project in a ventral to dorsal progression such that trigeminal fibers enter most ventral into the alar plate, followed by vestibular, mechanosensory lateral line and, if present, electroreceptive or auditory projections^1,10–12^. These central nuclei are molecularly distinct regions in the hindbrain. The most ventral trigeminal nuclei express ASCL1. Dorsal to that are the vestibular nuclei, which transiently express Neurog1, and the auditory nuclei, which express Atoh1^13,14^. Auditory and vestibular afferents project to their respective nuclei before central nuclei become post-mitotic^1^ and will project to the correct target region even if neither auditory nuclei nor hair cells ever develop^15^. This suggests that the presence of differentiated central target neurons is not necessary for proper auditory and possibly vestibular afferent guidance and rules out afferent attraction to and guidance by the target neurons themselves. Not only are inner ear afferents able to find their targets without central target differentiation, the specific hindbrain entry point also does not appear to affect pathfinding since transplantation of an additional ear rostral to the native ear in frogs resulted in projections of both ears to the vestibular nucleus^16^. Furthermore, ears transplanted near the spinal cord in frogs are able to navigate along dorsal tracts in the spinal cord continuous with the trigeminal tract. Once in the hindbrain those fibers reroute to the vestibular nuclei, making functional connections evaluated through behavioral and physiological assays^17^. Together these data suggest that molecular guidance cues play a role in central target pathfinding by inner ear afferents, though what the molecular nature of that guidance is and how conserved it is across vertebrates showing a rather stereotyped organization of afferents in the alar plate^18^ remains unknown. Several studies have shown through mutational analysis that certain transcription factors expressed in developing sensory neurons (Gata3^19,20^, Neurod1^21^) affect initial pathfinding properties. In addition, it has been shown that a component of the Wnt signaling pathway, Prickle1, affects pathfinding of spiral ganglion neurons^22^. Both Prickle1 and Wnt signaling receptors (Frizzled receptors) are regulated by Neurod1^23^, known to affect dorsoventral patterning of inner ear afferents^21^. Since inner ear afferents project to the same dorsoventral location within the hindbrains across all vertebrates^4,18^, we assume that all neural crest and placode derived cranial nerve afferents are navigating in the hindbrain using a conserved set of guidance molecules. Given the conservation of transcription factors and expression of diffusible molecules, such as Wnt, BMP, and Shh, across species and between the hindbrain and spinal cord^24,25^, we used a novel approach to investigate whether presumed conserved guidance molecules may play a role in the initial targeting of inner ear afferents through heterochronic, xenoplastic, and heterotopic transplantations in chickens and mice. We tested if these presumably conserved morphogens can guide mouse inner ear afferents in a chicken hindbrain despite over 300 million years of independent ear and brain evolution of these two lines of amniotes^26^.

Here we expand on our previous work on axon projection refinement in three-eared frogs by transplanting donor chicken otocysts rostral to the native ear. Donor ears were age-matched to the host (isochronic transplantations) or were younger than the host (heterochronic transplantations) to assess an effect of timing of afferent entry, as guidance cues may only be presented during a limited time of entry of the earliest arriving fibers only as suggested by timed arrival of various afferents^10–12^. To assess for possible conservation of molecular cues, we transplanted donor mouse otocysts (xenoplastic transplantation) rostral to the native chicken ear that were at comparable stage of development relative to the chicken host otocysts. Finally, since the pattern of gene expression and diffusible morphogen gradients in the hindbrain is also conserved with the spinal cord^15,27–32^, we transplanted donor chicken otocysts adjacent to the spinal cord (heterotopic transplantation) to ask whether afferents of transplanted ears navigate dorsally with or more dorsal to dorsal root ganglia projections in the spinal cord as they do in frogs^17^. This allows us to show further that afferents navigate using cues that are independent of the formation of vestibular or auditory nuclei, since these nuclei are absent in the spinal cord.

## Results

### Success of transplantation

We transplanted 164 chicken ears and 54 E9.5-10.5 mouse ears into 218 chicken hosts, adjacent to the native ear. Of these, 59 hosts that received chicken ears and 11 that received mouse ears survived for several days until fixation (about 30%). Most of the chicken hosts that did not survive were lost within the first 12 hours (about 66% of all deaths), likely due to complications from bleeding or healing during the transplant process. The remainder died leading up to collecting, either due to infection or improper closing of the amnion. Following our establishment that 3–5 days post-transplant was the optimal time for afferent projection of both ears into the hindbrain (see below), we later only collected these stages, leading to a slight increase in animal loss as we were not fixing animals before amnion closure or before infection occurred. Since most causes of death were due to excess bleeding and to a lesser extent to infection of the albumin or improper closing of the amnion around the chickens, rather than a defect in the chickens themselves, we do not expect a survivorship bias in our assessments.

**Figure 1:**
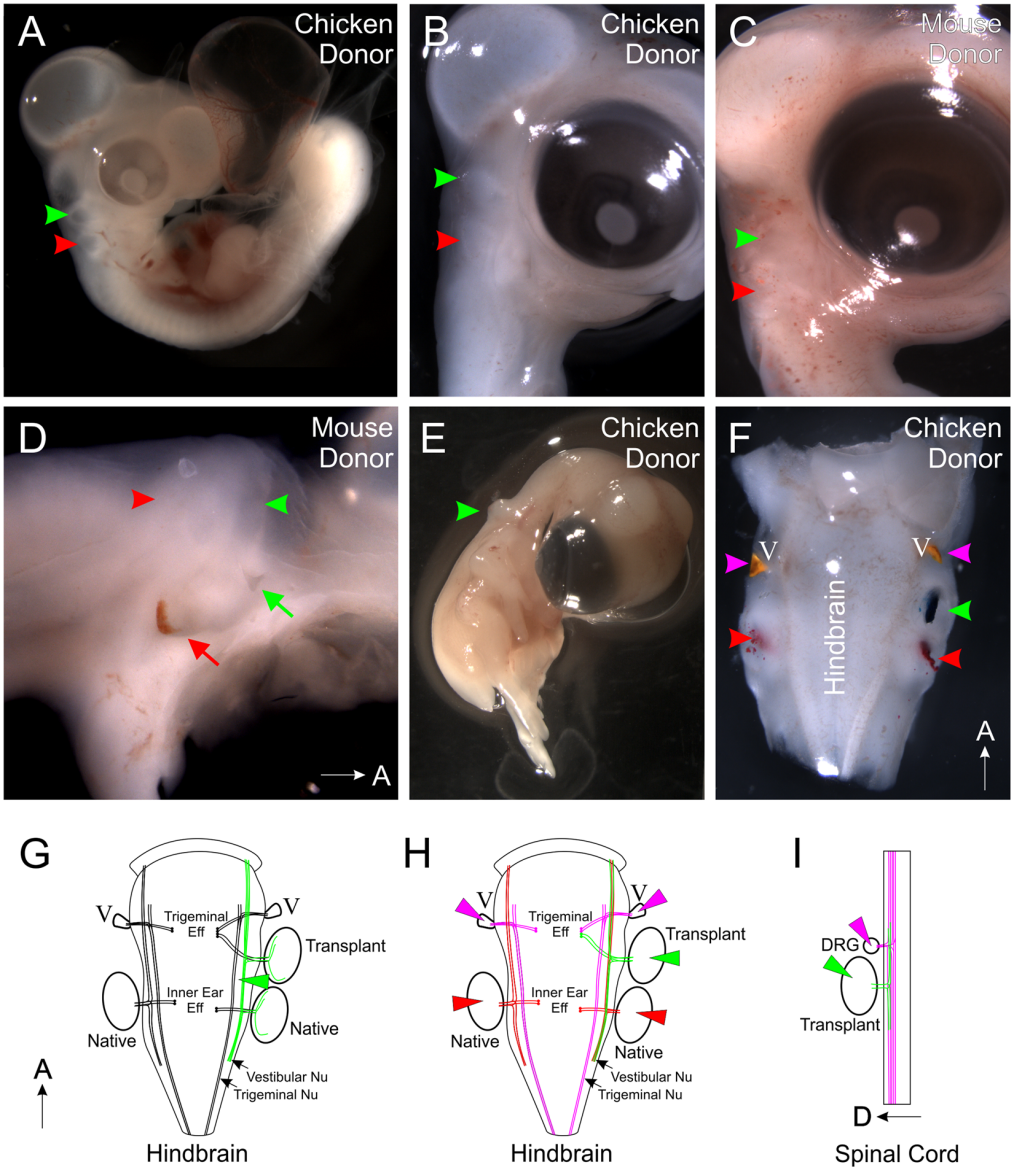
Transplantations and dye labeling. (**A**) Chicken embryo with a donor chicken ear (green arrowhead) rostral to the native ear (red arrowhead) following two days of incubation. **(B)** Chicken embryo with a donor chicken ear (green arrowhead) rostral to the native ear (red arrowhead) following four days of incubation. **(C)** Chicken embryo with a donor mouse ear (green arrowhead) rostral to the native ear (red arrowhead) following five days of incubation. **(D)** Side view of dissected chicken head revealing a second external opening (green arrow) adjacent to the transplanted mouse ear (green arrowhead). The native ear and external opening are marked in red. **(E)** Chicken embryo with a donor chicken ear (green arrowhead) transplanted caudally to the trunk adjacent to the spinal cord. **(F)** Dorsal view of dissected chicken head showing placement of lipophilic dye into the transplanted ear (green arrowhead), native ears (red arrowheads), and into the trigeminal ganglia (V, magenta arrowheads). **(G)** Diagram depicting placement of lipophilic dye into the dorsal alar plate (green wedge) and subsequent neurons that would be labeled (Note: in some instances, the trigeminal ganglion was also labeled given the close proximity of the trigeminal nucleus to the vestibular nucleus (See Fig. 2C)). (**H**) Diagram depicting placement of lipophilic dye into the native ears (red wedge), transplanted ear (green wedge) and trigeminal ganglia (magenta wedge) and subsequent neurons that would be labeled. **(I)** Diagram depicting the placement of lipophilic dye into the transplanted ear (green wedge) and into dorsal root ganglia (DRG, magenta wedge) and the subsequent neurons that would be labeled.

Successful transplantations were determined by the observed presence of an additional ear rostral to the native ear (Fig. 1). Of the 59 embryos receiving a chicken ear that survived until the designated fixation time, 54 had successful transplantations (Fig. 1A,B) and of the 11 embryos receiving a mouse ear that survived until fixation, all 11 had successful transplantations (Fig. 1C,D). In addition, we transplanted 38 chicken ears adjacent to the spinal cord. Of these, 13 survived until fixation and all 13 had successful transplantations (Fig. 1E). The best transplanted ears, where individual structures such as semicircular canals and cochlear/lagenar duct could be identified^26^ at the later stages of fixation or those that were comparable in development to the native ears, based on the timing of transplant, at earlier stages of fixation were selected for further analysis.

Interestingly, at the time of fixation, we observed a second external ear opening immediately adjacent to the rostrally transplanted ears of both chicken and of mouse (Fig. 1D, arrows), indicating that the inner ear may serve as an organizer for middle and external ear components of the ear. For this study, we did not follow up on this possibility as we focused on the central projection and the differentiation of inner ear.

### Transplanted ears develop beyond otocyst stage

**Figure 2:**
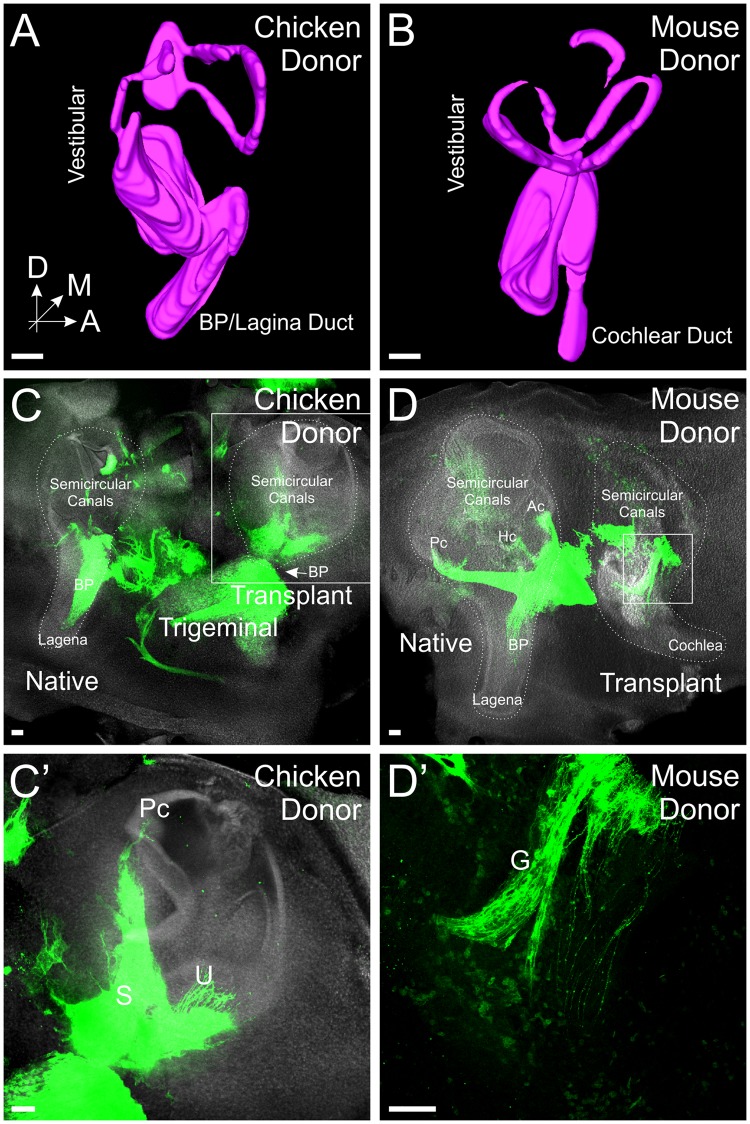
Transplanted ears develop beyond otocyst stage. (**A**) Three-dimensional (3D) reconstruction of Hoechst staining of a chicken ear three days after transplant. **(B)** 3D reconstruction of Hoechst staining of a mouse ear four days after transplant. **(C)** Lipophilic dye labeling (green) from the alar plate and Hoechst (white) staining of a transplanted chicken ear four days after transplant showing innervation of the ears. The basilar papilla (BP, arrow)/lagena grew perpendicular to the plane of the image and is therefore not visible in this image or in E. **(D)** Lipophilic dye labeling (green) from the alar plate and Hoechst (white) staining of a transplanted mouse ear four days after transplant showing innervation of the ears. Semicircular canals (Ac, anterior, Pc, posterior, Hc, horizontal) are labeled in the native chicken ear. **(C’)** Higher magnification of C (boxed area) showing innervation of the posterior canal (Pc) utricle (**U**) and saccule (**S**) from the transplanted ear. **(D’)** Higher magnification of D (boxed area) showing otic ganglia (**G**) adjacent to the mouse ear. Orientation for all panels is as in A (A, anterior, D, dorsal, M, medial). Scale bars represent 100 μm.

To determine the degree of development of the transplanted ears, ears were stained with Hoechst nuclear stain (n = 7 each for chicken and mouse) and subsequently 3D reconstructed^33^. While there was some variation in overall morphology, in general, transplanted ears of both chicken and mouse were found to have developed beyond the otocyst stage. Both transplanted chicken and mouse ears develop dorsal vestibular and ventral auditory components (Fig. 2A,B, S1A,B), as has been shown previously for development of transplanted ears in chicken^34^. In each specimen examined with Hoechst staining, we were able to observe semicircular canals as well as the lagenar/cochlear duct (Fig. 2A,B).

In addition, injection of lipophilic dye into the vestibular nucleus region of the alar plate of the hindbrain (Fig. 1G), as demonstrated in mice^35,36^, transganglionically labels the afferent innervation of the ears (n = 2 each for chicken and mouse; Fig. 2C-D’). Furthermore, this technique demonstrated otic ganglia adjacent to the developing ears (Fig. 2D’) that connected ears to the alar plate of the hindbrain consistent with known afferent and efferent connections in vertebrates^25,37^. Together these results show that in our ex-ovo culturing system, chicken and mouse ears do develop following transplantation, including inner ear ganglion neurons that project with their afferents to the hindbrain alar plate. However, while present at the later times of analysis, the auditory system in both transplanted chicken and mouse ears is not as completely developed as the vestibular, especially at some of the earlier stages we collected. Thus, for the remainder of our experiments, the dorsal vestibular portions of the ears were labeled with lipophilic dye and thus we are primarily looking only at vestibular projections for the remainder of the study.

### Inner ear afferents from transplanted chicken ears project to the vestibular nuclei regardless of timing or entry point

Ear transplantations were performed at stages after neurons have already begun delaminating from the host otocyst^38^. Donor ears were either age-matched or younger. The rationale for transplanting at these stages was that when the donor ears were from age-matched embryos, there would be a slight delay of hindbrain entry of afferents from the transplanted ear with respect to hindbrain entry of afferents from the native ear. When donor ears were from younger embryos, a larger delay in hindbrain entry between the two ears would occur. We first determined the time at which afferents from the transplanted ear could be labeled in the hindbrain (Table 1). In age-matched transplants, we labeled afferents from about half of transplanted ears following one or two days of incubation after transplant (n = 1/2 and 5/8, respectively), and always labeled afferents three days after transplantation. Furthermore, the animal in which afferents from the transplanted ear could be labeled in the hindbrain following one day of incubation had very little hindbrain projection compared with the native ears. In contrast, afferents from the native ears were consistently labeled both one and two days after transplant (n = 2/2 and 8/8, respectively). This suggests that there is indeed a slight delay between timing of entry between the two ears, even when the donor ear was at the same stage as that of the host.

**Table 1:** Timing of entry into the hindbrain.

Days Post Transplant	Transplanted Ear	Native Ear
1	1/2	2/2
2	5/8	8/8
3	2/2	2/2
4	2/2	2/2

Numbers represent animals in which dye-labeled inner ear afferents could be detected in the hindbrain.

**Figure 3:**
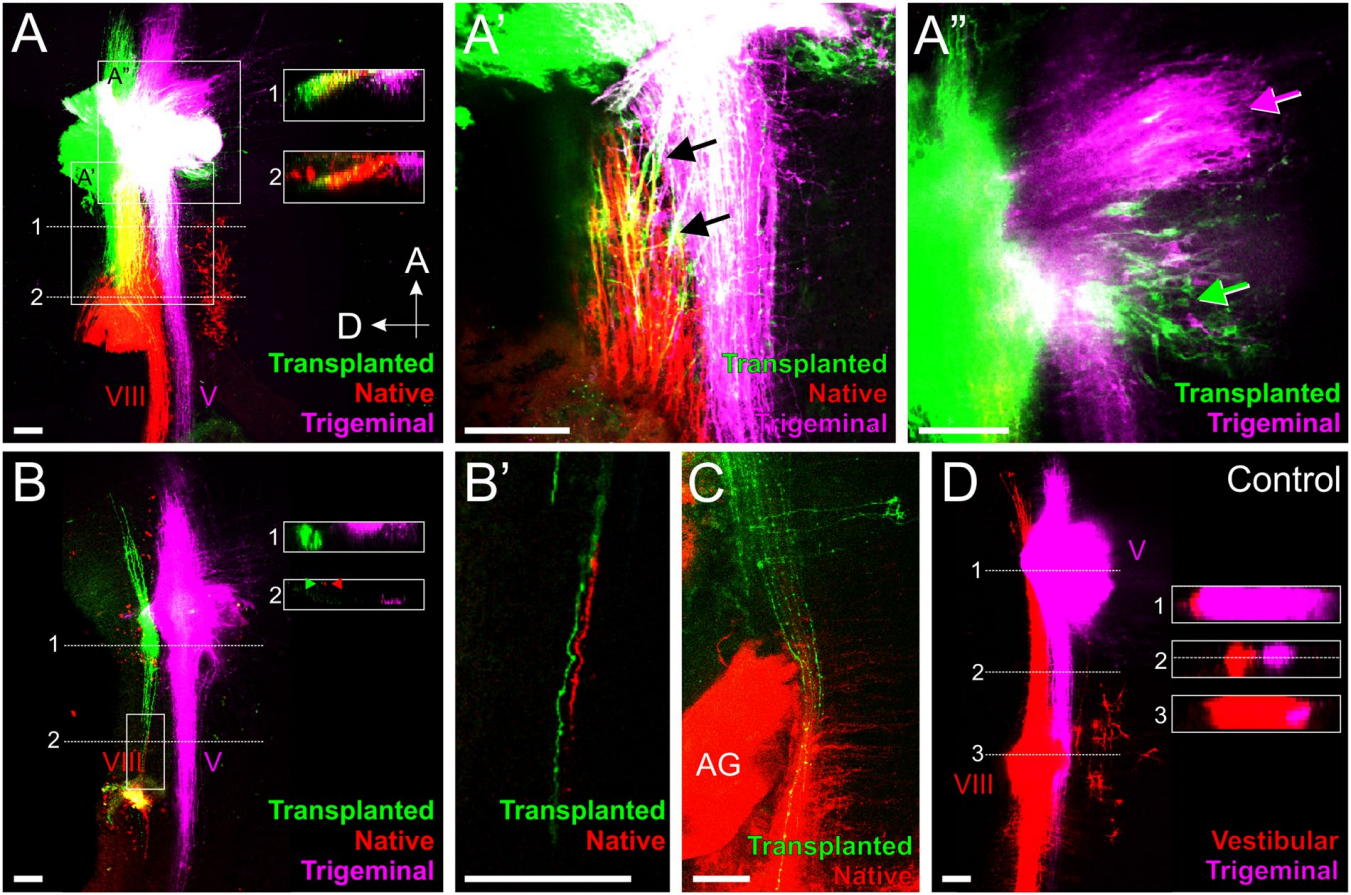
Afferents from transplanted chicken ears project to the vestibular nucleus regardless of timing or entry point. **(A)** Central projections of a transplanted chicken ear (green), native chicken ear (red), and trigeminal (magenta). Afferents from the transplanted ear enter with the trigeminal nerve (observed in 3/10 animals). Insets are single orthogonal sections through the z-dimension at the locations of the dotted lines in A. **(A’)** Single optical section of boxed area in A showing that inner ear afferents from the transplanted ear reroute from the trigeminal nucleus to the vestibular nucleus (arrows). **(A”)** Single optical section of the trigeminal motor nucleus in A showing, through backfilling of lipophilic dye, a subpopulation of trigeminal motor neurons projecting to the transplanted ear (green arrow). Magenta arrow designates motor neurons filled with trigeminal application of lipophilic dye. Animal in A-A” is 3 days post-transplant and age-matched. **(B)** Central projections of a transplanted chicken ear (green), native chicken ear (red), and trigeminal (magenta). Afferents from the transplanted ear enter separate from the trigeminal nerve (observed in 7/10 animals). Insets are single orthogonal sections through the z-dimension at the locations of the dotted lines in B. Arrowheads indicate position of single axons highlighted in B’. **(B’)** Single optical section of boxed area in B showing projection of afferents from the transplanted ear together with afferents from the native ear. Animal in B-B’ is 2 days post-transplant and is age-matched. **(C)** Central projections of a transplanted chicken ear (green) and native chicken ear (red) from an animal 4 days post-transplant with a heterochronic transplant. AG, auditory ganglia. **(D)** Single optical section of control projections of a native inner ear (red) and trigeminal (magenta) from an animal the equivalent of 2 days post-transplant. Insets are single orthogonal section through the z-dimension at the location of the dotted lines in D. Dotted line in inset 2 shows the location of the optical section in D. V, trigeminal nucleus; VIII, vestibular nucleus; Orientation for all panels is as in A (A, anterior, D, dorsal). Scale bars represent 100 μm.

To determine whether the timing of entry affects pathfinding, we transplanted donor chicken ears rostral to the native host ear (Fig. 1B) and at different time points. Using different colored lipophilic dye-soaked filter paper implanted into both the native and transplanted ears^36^, as well as the trigeminal ganglion (Figs. 1F, 1H), we found that afferents from the transplanted ears project to the vestibular nuclei from animals with age-matched donor ears at all stages examined that we could detect lipophilic-labeled afferents in the hindbrain (n = 8/8; Fig. 3A-B). In addition, we found afferents projecting to the vestibular nuclei in animals with ears transplanted from younger donors (HH 15–16 to HH 17–18; n = 2/2; Fig. 3C), suggesting that these slight delays in afferent entry into the hindbrain by a transplanted ear does not affect pathfinding, at least for the stages examined here. As a reference, we also implanted different colored lipophilic dyes into the native ear and trigeminal afferents on the side contralateral to the transplantation. Those tracings revealed direct projection of vestibular and trigeminal fibers to their respective nuclei in the hindbrain (n = 10/10, Fig. 3D), showing that under normal circumstances, inner ear afferents project directly to the vestibular nuclei adjacent and dorsal to trigeminal afferents. These data confirm previous work showing that afferents from different sensory organs project always directly to their specific nuclei, suggesting afferent specific attractive cues within the hindbrain^15,39^.

Upon closer examination of projections of transplanted ears, we observed that afferents from the transplanted ear either enter with their own entry point between the entry point of the native ear and that of the trigeminal nerve or enter together with the trigeminal afferents at the trigeminal entry point in Rhombomere 2. Afferents from the transplanted ear that entered with their own entry point (n = 7/10) invariably projected directly to the vestibular nucleus, together with afferents labeled from the native ear (Fig. 3B-B’). To our knowledge, this observation of inner ear neurons entering the hindbrain with their own entry point is the first time that the ability of inner ear afferents to digest their way through the neural crest derived meninges covering the developing hindbrain has been reported^3^. This ability is in line with such abilities of neural crest derived cranial nerve afferents, but contrasts to trigeminal branchial motor neurons that cannot leave the hindbrain in the absence of meningeal foramina generated by trigeminal afferents^40^. In contrast, if the afferents from the transplanted ear navigated along trigeminal afferents and entered together with them (n = 3/10), only some fibers immediately project dorsally to the vestibular nucleus together with afferents from the native ear and the remaining fibers project for a short distance with trigeminal afferents (Fig. 3A-A’). However, while the remaining fibers fasciculate initially with trigeminal afferents, they eventually segregated from the trigeminal afferents and rerouted into the vestibular nucleus between Rhombomeres 2 and 4 (n = 3/3; arrows, Fig. 3A’). These data suggest that transplanted inner ear afferents are molecularly attracted over short distances to the vestibular nucleus territory and this attraction can overcome the fasciculation with trigeminal afferents. Time lapse images of growing fibers are needed to reveal the details of choices made by navigating fibers to go beyond the suggestions derived from our stills that demonstrate the segregation from trigeminal fibers only after the fact.

### Transplanted chicken ears receive trigeminal branchial motor neuron innervation

In addition to labeling inner ear afferents, implantation of lipophilic dye into the transplanted ear revealed retrogradely filled basal plate branchial motor neurons^37,41^ within the trigeminal motor nuclei (Fig. 3A”), suggesting that these motor neurons reroute to the transplanted inner ear. Trigeminal branchial motor neurons labelled from the transplanted ear were observed in all animals in which the trigeminal motor population was labeled (n = 5/5). Furthermore, entry point of the transplanted ear afferents did not appear to affect the ability of trigeminal motor neurons to reroute to the transplanted ears. Labeled cell bodies in the trigeminal motor nucleus were found both in animals in which afferents from the transplanted ear entered the hindbrain with their own entry point (n = 3/5) and also in animals in which the afferents from the transplanted ear entered together with the trigeminal nerve (n = 2/5).

### Inner ear afferents from transplanted mouse ears project directly to the vestibular nuclei

**Figure 4:**
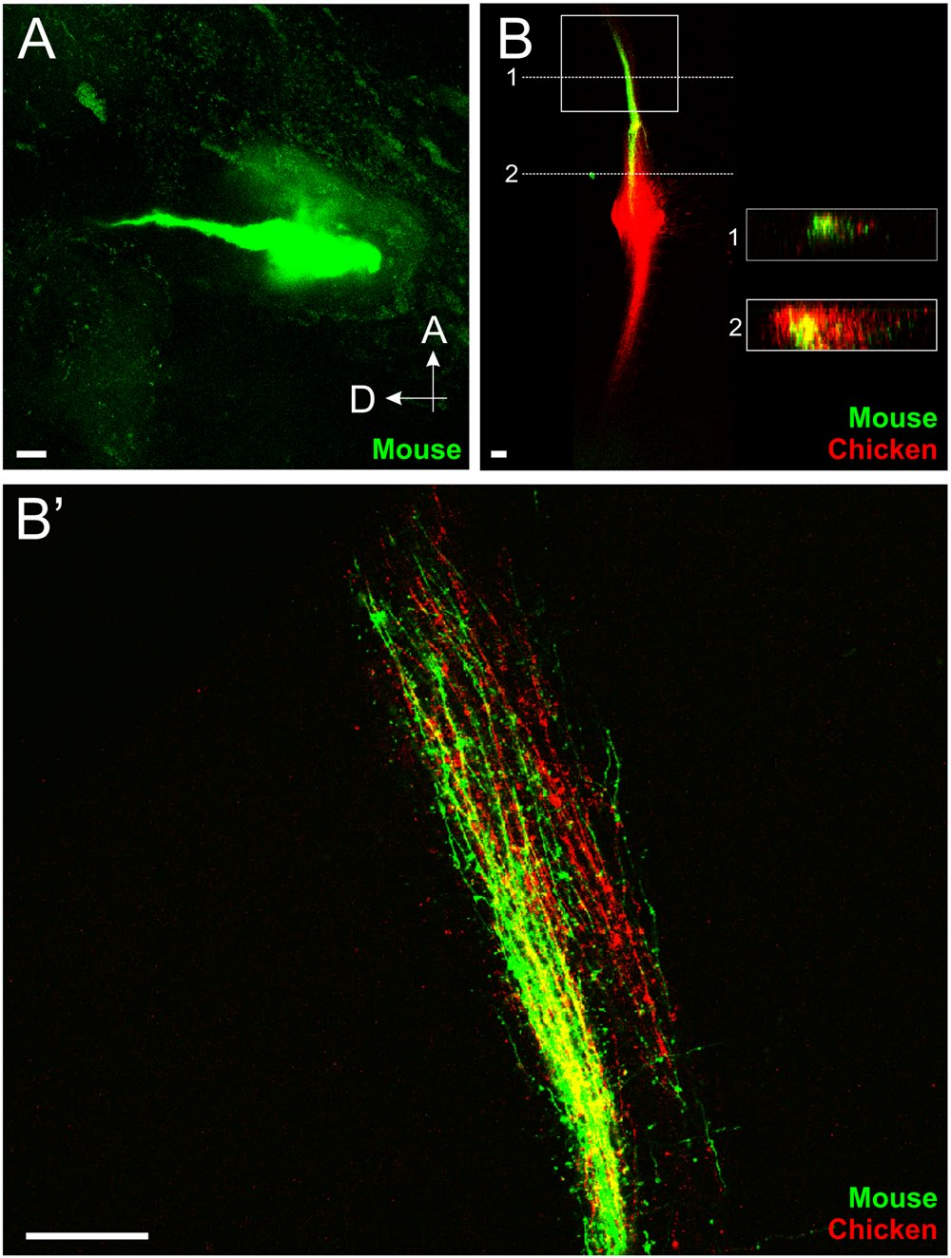
Afferents from transplanted mouse ears project to the vestibular nucleus of a chicken. (**A**) Lipophilic dye labeling of a transplanted mouse ear 5 days post-transplant showing directed projections of the afferents exiting the mouse ear (green) dorsally toward the hindbrain of the chicken. **(B)** Central projections of a transplanted mouse ear (green) and native chicken ear (red) 4 days post-transplant. Insets are single orthogonal sections through the z-dimension at the locations of the dotted lines in B. **(B’)** Single optical section of boxed area in B showing projection of afferents from the transplanted mouse ear together and in the same plane as afferents from the native chicken ear (observed in 4/4 animals). Orientation for all panels is as in A (A, anterior, D, dorsal). Scale bars represent 100 μm.

To determine whether inner ear afferents navigate using a set of guidance molecules that is conserved across amniote species, we transplanted mouse embryo otocysts rostral to the native ear of chicken embryos (Fig. 1C-D). Implantation of lipophilic dyes into the mouse ear revealed that mouse afferents projected directly towards the hindbrain (Fig. 4A). That the mouse afferents did not project in a random or disordered manner indicates that these mouse inner ear afferents are targeted directly toward the hindbrain of the chicken, comparable to transplanted chicken ears. Alternatively, this could indicate that only the afferents that reached the brain remained at the late stage when we analyzed them.

Comparison of central projections of afferents from the transplanted mouse ear with that of the native chicken ear through lipophilic dye labeling of both mouse and chicken ears revealed that mouse inner ear afferents projected to the chicken vestibular nucleus (n = 4/4, Fig. 4B-B’). Examination of single optical sections indicate that the mouse inner ear afferents project in the same dorsoventral column as native chicken inner ear afferents (Fig. 4B’). These data imply that mouse inner ear afferents are molecularly guided to the corresponding chicken nuclei, suggesting conservation of molecular guidance between those two amniote species that each evolved for over 300 million years from their last common ancestor.

### Inner ear afferents from ears transplanted to the spinal cord project dorsally even in the absence of any vestibular or auditory nuclei

**Figure 5:**
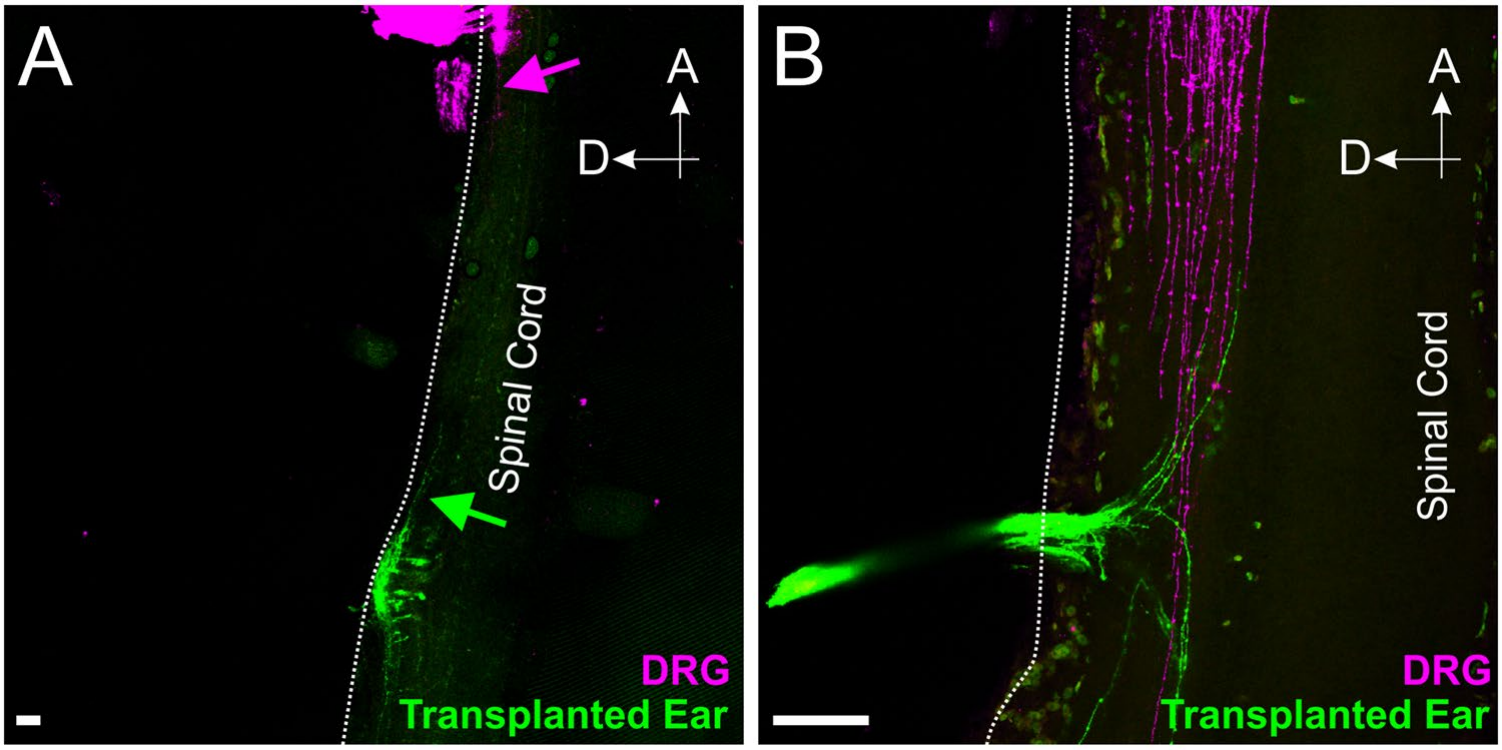
Afferents from transplanted chicken ears project dorsally in the spinal cord. (**A,B**) Lipophlic dye labeling of the transplanted ear (green) and dorsal root ganglia (magenta) from two different animals show that transplanted ear afferents project dorsally in the spinal cord. Arrows in A indicate projections of the transplanted ear (green arrow) and dorsal root ganglia (magenta arrow). Scale bar represents 100 μm.

Knowing that the pattern of gene expression and diffusible morphogen gradients in the hindbrain, which is conserved across species, is also conserved with the spinal cord^15,25,27–32^, we transplanted donor chicken otocysts to the trunk, adjacent to the spinal cord (Fig. 1D). Importantly, spinal cords develop neither vestibular nor cochlear nuclei thus allowing us to exclude additional attractions mediated from neurons of those nuclei. Implantation of lipophilic dye into the transplanted ear and into adjacent dorsal root ganglia (Fig. 1I) revealed that the transplanted ear projected in the most dorsal position of the spinal cord, at the same level or dorsal to the dorsal root ganglia projections (n = 5/5, Fig. 5A-B). Since dorsal root projections are consistent with the descending tract of V (trigeminal), this suggests inner ear afferents are targeted to a more dorsal position, as they are in the hindbrain^17^. These data, and those recently shown in frogs^17^, indicate that the inner ear afferents are possibly dorsally targeted by conserved molecular guidance cues across craniate chordates^32^, such as Wnts, BMPs and Lmx1a^25,31^, and not simply attracted to vestibular nuclei.

**Figure 6:**
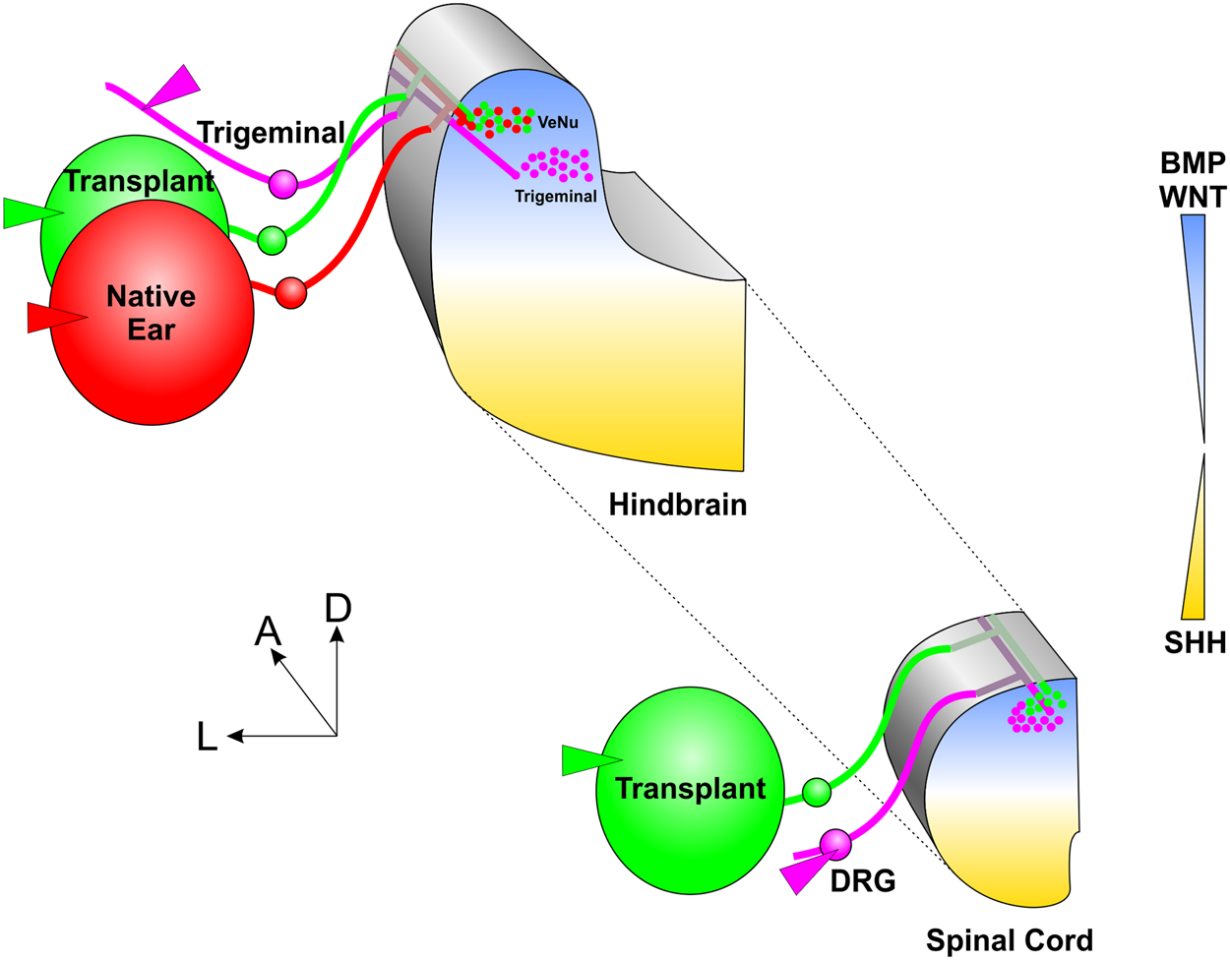
Schematic depicting the hypothesis that diffusible morphogens define the dorsoventral patterning of the hindbrain. Ears transplanted (green) adjacent to the native ear (red) project together in the region of highest expression of BMPs and Wnts (blue), and dorsal to trigeminal afferents (magenta). Similarly, when ears were transplanted to the spinal cord (green), afferents projected in the region of highest expression of BMPs and Wnts, at the level of or more dorsal-medial to dorsal root ganglia (DRG) afferents (magenta). We predict that inner ear afferents are targeted by diffusible morphogens expressed in the hindbrain and spinal cord.

In summary, our transplantation expand previous work on *Xenopus*^16,17,42,43^, demonstrating that transplanted ears of chicken and mice target primarily dorsal areas of hindbrain and spinal cord. The dorsal spinal cord projection in the absence of vestibular or auditory nuclei formation supports that inner ear afferents orient using diffusible molecules setting up dorsoventral gradients to pattern hindbrain and spinal cord (Fig. 6) and are not attracted by molecules released from vestibular nuclei themselves, expanding our observation on auditory afferent targeting in the absence of cochlear nuclei^15^.

## Discussion

The results presented here expand on previous studies in creating quail-chicken and mouse-chicken hybrids that have shown that various cross-species transplantations can work^44–49^ indicating conservation of levels of molecular developmental signaling at least across amniotic vertebrates. Here we show that not only can a xenoplastic tissue, the mouse ear, develop in a chicken, but also show for the first time how neurons emanating from the transplanted mouse ear^2,3^ can navigate in the chicken hindbrain to home selectively to the chicken vestibular nucleus as identified by the overlap with native ear projections. Furthermore, our results expand on prior work specifically on ear transplantations that have shown that neurons from transplanted ears can reach the central nervous system (CNS)^42,43^ and once in the CNS, have the ability to find the vestibular nucleus^16,17,43^ and can influence the dendritic branching of second-order neurons^50,51^. Our data imply conservation of candidate guidance molecules across amniotes and between hindbrain and spinal cord.

Afferents from a transplanted ear can navigate to the vestibular nucleus overlapping with native inner ear afferents, even with a delay of hindbrain entry with respect to the native ear. This suggests that inner ear afferents are molecularly targeted to the vestibular nucleus, rather than by simple timing of entry of the various hindbrain afferents as has been previously suggested^10,12^. However, the data observed in axolotl^10^ and zebrafish^12^ that had suggested that afferent central targeting is based on timing may be instead the result of the timing of expression of the molecular cue(s) themselves. Alternatively, molecular cue(s) are expressed for a longer duration of time and different afferents are each targeted to a particular gradient. The time window during which inner ear afferents can properly navigate remains unknown and requires further testing by increasing the delay of transplantation. Such information is crucial for future attempts to improve cochlear implant function through sensory neuron seeding in the partially depleted Rosenthal’s canal of deaf mammals, including humans that need to establish topologically correct connections of these transplanted neurons with the brain to be beneficial.

Our data in which inner ear afferents rerouted into the vestibular nucleus following fasciculation with trigeminal afferents for a short distance in animals in which the afferents entered together with the trigeminal afferents also supports the hypothesis that inner ear afferents are molecularly targeted to the dorsal alar plate of the hindbrain. Furthermore, this targeting of inner ear afferents to the vestibular nucleus overcomes cell-cell interactions from fasciculation with trigeminal afferents. Such rerouting to the vestibular nucleus by inner ear afferents from the trigeminal nucleus was occasionally observed when ears were transplanted rostrally to the orbit^43^. In addition, functional rerouting of afferents to the vestibular nucleus was observed when the ear was transplanted adjacent to the spinal cord^17^. Together, these results suggest that once in the hindbrain, inner ear afferents can defasciculate from other fibers and target vestibular nuclei.

Projections to the vestibular nucleus of the chicken from mouse inner ear afferents or projections to the most dorsal aspect of the spinal cord from adjacently transplanted ears provides support for a common molecular guidance factor that is conserved not only between amniote species but also between the hindbrain and spinal cord. The dorsoventral gradients of Wnt, bone morphogenetic protein (BMP) and Sonic hedgehog (Shh) depend on the dorsally expressed Lmx1a/b^25,52^ and are conserved between species and between the hindbrain and spinal cord^15,25,27–32^. These conserved expression profiles are thus likely candidates for molecular guidance that provide a stereotyped pattern of innervation^10,11^. Consistent with the hypothesis that inner ear afferents might use diffusible molecules for central pathfinding, is that mutations in the Wnt-planar cell polarity pathway component, Prickle1, show aberrant central projections of inner ear afferents^22^. Furthermore, the dorsal Wnt gradients that are conserved between the hindbrain and spinal cord are not conserved with the midbrain due to absence of Lmx1b expression in that brain region that guides Wnt expression^25^. This may explain the random and inconsistent projections of inner ear afferents entering the midbrain with the oculomotor or trochlear nerve when transplanted to the orbit^43^, as opposed to the targeted projections to the vestibular nucleus within the hindbrain observed here in chicken and mice and previously in frogs^16,17^.

In addition to afferent projections, we also observed evidence of efferent innervation of transplanted ears by backfilling trigeminal branchial motor neurons located in the hindbrain basal plate^25,37,41^ from dye implanted into transplanted ears. Previously, we have shown that ears transplanted caudally to the trunk to replace a somite or further rostrally to the orbit to replace the eye can be innervated by spinal motor neurons or by oculomotor and trochlear motor neurons, respectively^42,43^. Transplantation of an ear adjacent to trigeminal branchial motor neurons are additional support that any motor neuron can become an efferent to the ear, should the ear be placed in their axonal trajectory. This further supports the hypothesis that inner ear efferents arose from rerouted facial branchial motor neurons when the ear evolved in ancestral craniates in the place of somite-derived muscle tissue^37,53,54^.

In conclusion, the data presented here demonstrate that inner ear afferents are targeted to their specific nuclei within the hindbrain likely by yet unidentified molecule(s) and that these molecules are present for some period of time, conserved across amniote species, and conserved between the hindbrain and spinal cord. Evidence presented here and in recent papers on other tetrapods^16,17,42,50^ implicate that the molecular gradients associated with dorsoventral patterning of the hindbrain and spinal cord, such as Wnt signaling^22^, may be involved in inner ear afferent central pathfinding. Future directions are to identify these molecules and investigate their role in guiding inner ear afferent central projections through a combination of molecular manipulations and transplantations.

## Methods

### Animals

All animal work was conducted according to the Care and Use of Laboratory Animals and procedures were approved by the University of Iowa Institutional Animal Care and Use Committee (IACUC) (ACURF #1103057).

Fertilized chicken eggs were obtained from Hoover’s Hatchery (Rudd, IA) and Aleta’s Eggs (Belle Plaine, IA) and kept at 18 °C until incubation (maximum of 1 week at 18 °C). Eggs were incubated at 37 °C at 70% humidity for approximately 4 days prior to transplantation. Under our conditions, chicken embryos were between Hamburger-Hamilton (HH) stages 14–18^55^ at the time of transplant, though most transplantations were performed between stages 16–18.

Wild type mouse embryos were obtained from pregnant females at embryonic day (E) 10.5. Pregnant females were anesthetized by injection of a lethal dose of Avertin (1.25% of 2.2.2-tribromoethanol at a dose of 0.025 ml/g of body weight) and decapitated. Uterine horns containing the mouse embryos were removed from the females and processed as described below.

### Eggless culture and ear transplantation

For our eggless culturing technique, we followed the general protocol of Cloney and Franz-Odendaal^56^. Eggs were wiped with 70% ethanol and kept on their sides prior to cracking. Eggshells were cracked ventrally by tapping them against the narrow blunt end of a histological knife held securely in a microtome holder. Cut eggs were gently opened at the ventral cut and the content was decanted into a sterile weigh boat (88 × 88 × 23 mm; Fisher Scientific, catalog #08732113). Chickens were inspected for integrity of the yolk and only those with intact yolk were used as ear recipients. Those with ruptured yolks became ear donors.

For donors: For donor chickens, embryos (HH stage 14–18) were carefully cut free of the yolk with sterile dissection scissors and placed in sterile tissue culture medium (DMEM with LGlutamine, Fisher Scientific). For donor mice, embryos (E9.5–10.5) were carefully removed from the uterine horns and placed in sterile tissue culture medium (DMEM with LGlutamine, Fisher Scientific). For chicken and mice, the left and right otic vesicles were dissected out with sterile tungsten needles and placed in sterile tissue culture medium in a dish on ice for later transfer into a host. Dextran amine dye (Texas Red 3000 MW, Molecular Probes) or methylene blue (Sigma) was added to the tissue culture medium to temporarily label the ears for ease of identification during transplantation. Time delay between ear removal from donor and implantation into host varied between 5–90 minutes with no apparent effect on donor ear development.

For hosts: The amnion around the HH stage 14–18 chicken embryo’s head or torso was carefully opened with sterile forceps. A single otic vesicle (from chicken or mouse) was removed from the dish on ice and transferred to the host. For transplants adjacent to the hindbrain, the skin rostral to the native right ear was opened using sterile tungsten needles and the donor otic vesicle was pushed inside. For transplants adjacent to the spinal cord, the skin next to the spinal cord at the level of the forelimb bud was opened and the donor otic vesicle was pushed inside. Care was taken to orient the otocyst so that the endolymphatic duct pointed dorsal to avoid formation of enantiomorphic twins through rotation of ear axis relative to body axis^57^.

A sterile Plexiglas lid was affixed to the weigh boat with scotch tape, 40 µl Penicillin/Streptomycin (5,000 units penicillin, 5 mg streptomycin per ml; Sigma, catalog #P4458) was added to the albumin, and the embryo culture was placed in the 37 °C incubator for an additional 1 to 5 days. The time of transplant as well as the stage of the host and the stage of the donor were recorded. Following the additional 1 to 5 days of incubation, embryos were fixed with 4% paraformaldehyde (PFA) through either immersion-fixation (1–2 day incubation) or through cardiac perfusion using a peristaltic pump followed by immersion (3–5 day incubation). Embryos were fixed at least overnight in 4% PFA before further processing.

### Lipophilic dye labeling

Prior to injecting lipophilic dye, for some animals, heads were removed and hemisected to reveal the hindbrain. Small pieces of lipophilic dye-soaked filter paper (NeuroVue™, (Polysciences, Inc.)^58,59^ were flattened and implanted into the region of the vestibular nucleus in the alar plate between the native and transplanted ears (NeuroVue™ Maroon) to label afferents of the native and transplanted ears (Fig. 1G). In other animals, heads were removed from the fixed embryos and tissue carefully dissected away to reveal the inner ears. Lipophilic dye was implanted dorsally into the native (NeuroVue™ Red) and transplanted ears (NeuroVue™ Maroon) as well as into the trigeminal nerve (NeuroVue™ Jade) (Fig. 1F,H), either into the whole dorsal portion of the ear or into select vestibular sensory epithelia in the dorsal portion of the ear. On the contralateral/control (left) side of the animal, lipophilic dyes were implanted dorsally into the native ear (NeuroVue™ Red) and into the trigeminal (NeuroVue™ Jade) (Fig. 1F), either into the whole dorsal portion of the ear (for animals 2 days post-transplant or younger) or into select vestibular sensory epithelia in the dorsal portion of the ear (for animals 3 days post-transplant or older). Thus, with these placements of lipophilic dye, we should primarily label vestibular, rather than auditory, neurons. For ears transplanted to the spinal cord, skin was removed around the transplanted ear and lipophilic dyes were implanted into the transplanted ear (NeuroVue™ Maroon) and adjacent dorsal root ganglia (NeuroVue™ Red) (Fig. 1I).

Animals were placed in vials in 0.4% PFA and incubated at 60 °C for 2–5 days. For the chicken and mouse ears labeled from the alar plate, Hoechst nuclear stain was added to the vials for the duration of the incubation. Dye diffusion was monitored daily with a fluorescent microscope to ensure proper diffusion over the distance. Ears or brains were mounted on a slide in glycerol. Brains were either whole-mounted or hemisected. Images were taken with a Leica TCS SP5 confocal microscope using Leica LIS software. It has been show previously in mice and chicken that tracing afferents can serve as a proxy to identify vestibular nuclei or the vestibular cerebellum^11,35,60^, thus we used labeled afferents from the native ear to identify the vestibular nucleus in the alar plate. Positive innervation by afferents from the transplanted ear was defined by at least one labeled axon projecting into the vestibular nucleus.

### Three-dimensional reconstruction

Transplanted ears from chicken and mouse were stained with Hoechst nuclear stain overnight. Ears were mounted in glycerol on a microscope slide. Confocal z-series images at 3 µm were taken of the ears using a Leica TCS SP5 confocal microscope. Z-series stacks were loaded into Amira Version 5.4 software for manual segmentation as described previously^33^.

## Electronic supplementary material

Supplementary Dataset 1

## Data Availability

The data that support the findings of this study are available from the corresponding author upon reasonable request. Tables of raw data are included as supplemental material.
