# Supplementary material for: Ear transplantations reveal conservation of inner ear afferent pathfinding cues
Source: Sci Rep. 2018 Sep 14;8:13819. doi: 10.1038/s41598-018-31952-y (PMC6138675; doi:10.1038/s41598-018-31952-y)
Supplement: Supplementary file 1 — Supplementary Dataset 1 [file 41598_2018_31952_MOESM1_ESM.pdf]

Ear transplantations reveal conservation of inner ear afferent pathfinding cues

Karen L. Elliott* and Bernd Fritzsch

Department of Biology, University of Iowa, Iowa City, IA 52242

Author for correspondence:

Karen L. Elliott, Ph.D.

Department of Biology
University of Iowa
Iowa City, IA, 52242, USA
karen-elliott@uiowa.edu

Tel: 319-335-1089
Fax: 319-335-1069

Table S1. Individual transplantation data for central projections from rostrally transplanted ears.

Animal	Transplant	Age of Donor	Days Post Transplant	Vestibular Projections	Enter on Own	Enter with Trigeminal
1	Chicken	Same as host	1	Yes	Yes	No
2	Chicken	Same as host	1	No	N/A	N/A
3	Chicken	Same as host	2	No	N/A	N/A
4	Chicken	Same as host	2	Yes	Yes	No
5	Chicken	Same as host	2	Yes	Yes	No
6	Chicken	Same as host	2	No	N/A	N/A
7	Chicken	Same as host	2	Yes	Yes	No
8	Chicken	Same as host	2	Yes	No	Yes
9	Chicken	Same as host	2	Yes	Yes	No
10	Chicken	Same as host	2	No	N/A	N/A
11	Chicken	Same as host	3	Yes	No	Yes
12	Chicken	Same as host	3	Yes	No	Yes
13	Chicken	Same as host	4	Yes	Yes	No
14	Chicken	Younger than host	4	Yes	Yes	No
15	Chicken	Younger than host	4	Yes	Yes	No
16	Mouse	E10	5	Yes	N/A	N/A
17	Mouse	E9.5	5	Yes	N/A	N/A
18	Mouse	E10	5	Yes	N/A	N/A
19	Mouse	E10	5	Yes	N/A	N/A

Vestibular projections identified as positive based on the presence of at least one labeled axon from the transplanted ear. Additional animals were used for Hoechst staining, 3D reconstructions, and hindbrain dye injections (n = 7 each for animals with chicken donor or mouse donor ears).

Table S2. Individual transplantation data for ears transplanted adjacent to the spinal cord.

Animal	Days Post Transplant	Dorsal Projections
1	5	Yes
2	5	Yes
3	5	Yes
4	5	Yes
5	5	Yes

Figure S1. Transplanted ears develop beyond otocyst stage. (A) Three-dimensional (3D) reconstruction of Hoechst staining of a chicken ear three days after transplant. **(B)** 3D reconstruction of Hoechst staining of a mouse ear four days after transplant.

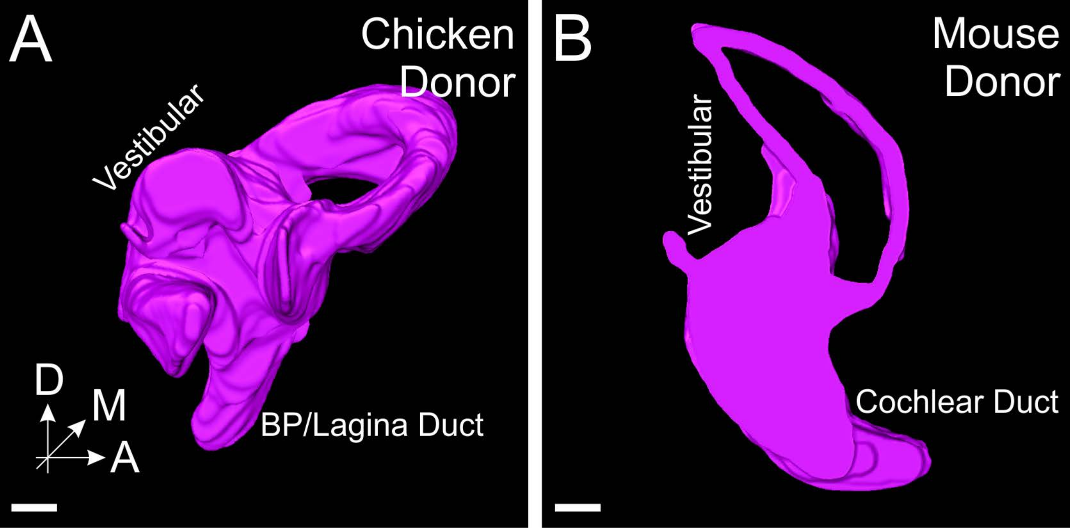
A
Chicken
Donor
Vestibular
D
M
A
BP/Lagina Duct
B
Mouse
Donor
Vestibular
Cochlear Duct
